# Zirconium 89 and Copper 64 for ImmunoPET: From Antibody Bioconjugation and Radiolabeling to Molecular Imaging

**DOI:** 10.3390/pharmaceutics16070882

**Published:** 2024-06-30

**Authors:** Laure Badier, Isabelle Quelven

**Affiliations:** Toulouse NeuroImaging Center (ToNIC), INSERM/UPS UMR 1214, University Hospital of Toulouse-Purpan, CEDEX 3, 31024 Toulouse, France; laure.badier@inserm.fr

**Keywords:** immunoPET, antibody, chelators, radiochemistry

## Abstract

Immunotherapy has transformed cancer treatment. Nevertheless, given the heterogeneity of clinical efficacy, the multiplicity of treatment options available and the possibility of serious adverse effects, selecting the most effective treatment has become the greatest challenge. Molecular imaging offers an attractive way for this purpose. ImmunoPET provides specific imaging with positron emission tomography (PET) using monoclonal antibodies (mAb) or its fragments as vector. By combining the high targeting specificity of mAb and the sensitivity of PET technique, immunoPET could noninvasively and dynamically reveal tumor antigens expression and provide theranostic tools of several types of malignancies. Because of their slow kinetics, mAbs require radioelements defined by a consistent half-life. Zirconium 89 (^89^Zr) and Copper 64 (^64^Cu) are radiometals with half-lives suitable for mAb labeling. Radiolabeling with a radiometal requires the prior use of a bifunctional chelate agent (BFCA) to functionalize mAb for radiometal chelation, in a second step. There are a number of BFCA available and much research is focused on antibody functionalization techniques or on developing the optimum chelating agent depending the selected radiometal. In this manuscript, we present a critical account of radiochemical techniques with radionuclides ^89^Zr and ^64^Cu and their applications in preclinical and clinical immuno-PET imaging.

## 1. Introduction

Over the last 30 years, monoclonal antibodies (mAbs) have allowed major advances in the treatment of cancers. These improvements have been mainly possible by advances in understanding of immune mechanisms and technological developments in the production of mAb [1,2]. Immune checkpoint blockade and Fc-mediated antibody-dependent cellular cytotoxicity (ADCC), antibody-dependent cellular phagocytosis (ADCP) and complement-dependent cytotoxicity (CDC) are the main mechanisms of antibody-based immunotherapy. Many new immune checkpoint molecules have been identified, such as PD-1/PD-L1 or CTLA-4, conducting to interesting target. Technological advances allowing production of chimeric, humanized or human antibodies have reduced mAb immunogenicity. In addition to the traditional mAbs, optimized mAbs have been developed to enhance their therapeutic properties. By improving the interactions between the Fc fragment and the FcRs and by optimizing the glycosylation and amino acid sequence of the Fc, more efficient ADCC can be achieved. To overcome the limitations of mAbs size, including slow kinetics and potentially low penetration into large tumors, Ab fragments, defined by the absence of the Fc moiety and by their smaller size and high affinity, have been developed [3]. More recently, development of bispecific mAbs has opened up new opportunities in immunotherapy, such as the recruitment of cytotoxic effector cells from the immune system to target pathogenic cells to enhance effector cell activation and anti-tumor immunotherapy [4]. Additional properties can be added to mAbs by coupling them to agents such as drugs, enzymes (abzymes) or radioisotopes [5].

The main advantage of immunotherapy is its specific action and its use in a wide range of potential indications [3]. Approximately 1200 mAbs are currently in clinical trials and approximately 200 are under regulatory review or approval [6]. Nevertheless, given the heterogeneity of clinical responses to immunotherapy and the wide range of therapeutic options available, a major challenge has emerged: personalizing patient care [7,8]. In recent years, the notion of theranostic has gained ground in the literature. This concept lies at the frontier diagnosis and therapy with the aim of selecting patients who will respond to specific treatments and improving patient care through personalized medicine. The theranostic concept has two aspects. The first involves using a vector, initially coupled to a radionuclide for diagnostic purposes, to study the molecular expression of the disease. Once this expression has been documented and validated, the same vector can be used, this time coupled with a therapeutic radionuclide, to provide targeted treatment of the tumor. The second aspect of theranostics is a broader concept. It is based on the radiolabeling of a molecule of interest in order to image its biodistribution and binding to its target, and thus to predict individual therapeutic efficacy. Such theranostic approaches are necessary to improve treatment efficiency, but also necessary in view of the high cost of this type of treatment.

Non-invasive imaging represents a powerful tool to investigate mAbs behavior and potency. Positron emission tomography (PET) is a widely used molecular imaging technology, particularly developed in clinical oncology. Thanks to vectors radiolabeled with β^+^ emitting radionuclide, PET allows non-invasive study of the expression of molecular targets. Different vectors can be used to deliver the radionuclide to the target of interest, allowing the visualization of organ function or the molecular expression on the surface of cells [9]. Among the radiotracers used for PET imaging, the most widely used radiotracers is 2-Deoxy-2-(^18^F)fluoro-D-glucose or fluorodeoxyglucose (^18^F-FDG), which is indicated in staging, restaging, detecting recurrences, and predicting the prognosis of various cancers [10,11]. Although ^18^F-FDG is still a key radiotracer in oncology, due to its high sensitivity and a wide range of indications, ^18^F-FDG is not specific to tumor cells, and ^18^F-FDG uptake is also observed in inflammatory or infectious lesions. More specific radiotracer could be interesting to investigate treatment responses and efficacy. 

**Figure 1:**
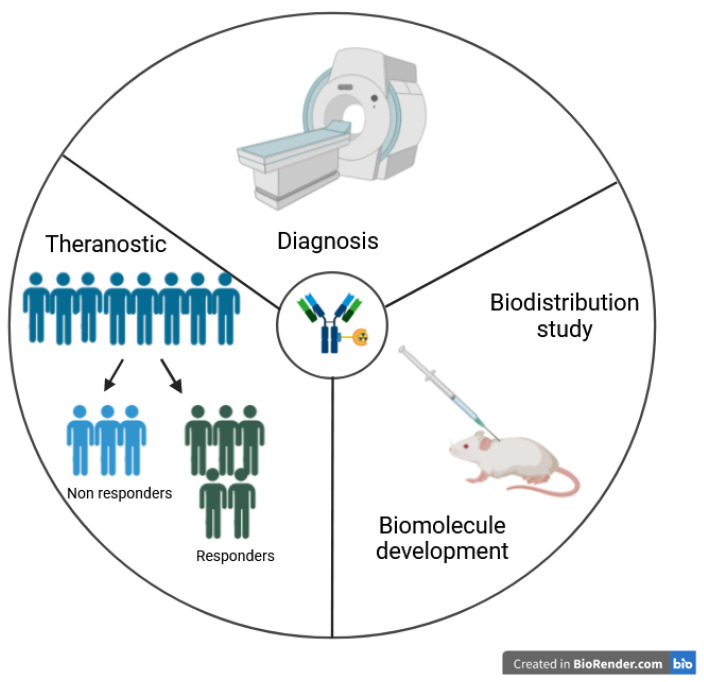
ImmunoPET applications.

In this way, mAbs, which specifically recognize antigens on pathologic cells, represent promising vectors. ImmunoPET is a paradigm-shifting molecular imaging modality combining the specific targeting ability of mAb and the inherent sensitivity of PET technique. This non-invasive method could quantitatively assess in vivo the target expression and distribution in the whole-body. ImmunoPET imaging enables the study of drug target expression by quantifying tracer uptake into the tumor, provides data to support drug development [12] and can help with patient selection, stratification and response monitoring [9]. This technique may allow the development of new theranostic tools, i.e., patient stratification, diagnosis, selection of targeted therapies, assessment and prediction of treatment response and adverse effects [12,13]. Based on immunoPET, treatment strategies could be tailored for individual patients before administering expensive and potentially toxic therapies. A large number of preclinical and clinical studies highlight the growing importance of immunoPET as a diagnostic and theranostic tool in oncology. Furthermore, immunoPET can be used in preclinical studies for biomolecule development (Figure 1).

**Figure 2:**
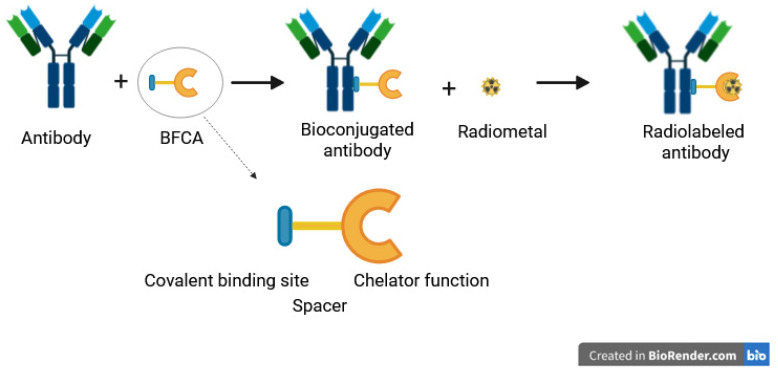
Antibody bioconjugation using a bifunctional chelate agent (BFCA) and radiolabeling with metallic radionuclide.

Vector radiolabeling for molecular imaging requires a good match between the vector kinetics and the half-life of the radioelement. When using whole mAb, characterized by a high molecular weight (150 kDa) and a slow kinetic, it is necessary to use radioelements with a relatively long half-life. Radioisotopes with short half-lives, such as ^18^F (t_1/2_ = 110 min) or ^68^Ga (t_1/2_ = 67.7 min), which are common in clinical practice, have the advantage of low radiation exposure. However, they are not optimal for long circulating probes, such as mAbs. Therefore, radiolabeling with long-lived radioisotopes such as ^124^I (t_1/2_ = 4.2 days), ^64^Cu (t_1/2_ = 12.7 h), and ^89^Zr (t_1/2_ = 3.3 days) is required for the better assessment of the biodistribution of such tracers [14]. In this review, we focus on ^64^Cu and ^89^Zr, both of which are radiometals and involve chelation chemistry. ^64^Cu and ^89^Zr are gradually becoming the gold standard for immunoPET. As ^64^Cu and ^89^Zr are radiometals, radiolabeling involve mainly indirect techniques with a bifunctional chelate agent (BFCA) which need to be conjugated to the biomolecule before radiolabeling. BFCA are composed of a chemical function enable to coupling an antibody and a prosthetic group to coordinate a radiometal (Figure 2). 

Chemical function of the BFCA must allow conjugation to the biomolecule under reaction conditions compatible with the latter (e.g., pH and temperatures) [15]. The choice of chelating group depends on the radioactive metal and its coordination properties. In particular, the denticity of a ligand, which is defined by its ability to complex a central atom via a number of bonds, influences the stability of the metal complex. Moreover, complexes are situations of chemical equilibrium, meaning that the association can be reversible. Several factors influence stability. First, the thermodynamic factor, which represents the strength of the Lewis acid/base combination, characterizes the equilibrium of the system. Secondly, the kinetic factor, which is proportional to the energy required to dissociate the complex. Another factor, lability, is defined by the rate of complexation-decomplexation. It is characterized by the rate at which the water molecules in the complexes are exchanged. Metal contamination, pH and temperature of the reactive environment can also influence the stability of the complex. Some chelating agents can be used in combination with different radiometals as DOTA (2,2′,2″,2‴-(1,4,7,10-tetraazacyclododecane-1,4,7,10-tetrayl)tetraacetic acid), but there are often optimized chelating systems tailored to specific radiometals. Much research has been devoted to the development of functionalization techniques and to optimization of chelating agents. Recently, the major challenge has been to propose site-specific bioconjugation to prevent mAb affinity impairment. In vivo pre-targeting strategies are also in development. The idea is that mAb and radioelement are separately injected and will combine within the body. These techniques need system allowing recognition of both entities in vivo. These strategies are mainly used for radiotherapy or with short-lived radionuclides because they decrease the circulation time of the radioactivity, reduce the uptake of the radionuclide in healthy nontarget tissues, and facilitate the use of short-lived radionuclides [16]. In this review, pre-targeting strategies will not be discussed, because they are not widely used for immunoPET with ^64^Cu and ^89^Zr.

In this review, we will firstly, describe developments in ^64^Cu and ^89^Zr radiochemistry, including antibody bioconjugation methods and chelators available. We will focus on antibody-based radiotracers, but the developments described are also relevant to other types of vectors, notably antibody fragments, nanobodies or affibody molecules. Finally, we will highlight recent preclinical and clinical applications of radiolabeling of mAb with ^89^Zr and ^64^Cu.

## 2. Antibody Bioconjugation Methods

Indirect antibodies radiolabeling with radiometals requires BFCA, which consist of a ligand that allows metal chelation and a functional group that allows covalent binding to the vector. Functionalization techniques can be separated in random labeling methods and site-specific techniques like cys engineering and click chemistry. The description of the different bioconjugation strategies is not exhaustive, as there are many different ways of labeling mAb.

Before describing the different techniques, it should be noted that some precautions are required for antibodies bioconjugation. Firstly, the conjugation conditions must respect the tertiary and quaternary antibody structure, e.g., the reaction temperature must not exceed 50 °C to avoid antibody denaturation or the pH must be kept between 7 and 9 [17,18]. The covalent bond between chelator and protein must be stable in vivo. In addition, the antibody-chelating agent complex must have the same in vivo properties as the antibody alone [19]. As chelator fixation on antibody can alter antibody pharmacokinetics and affinity, it is important to study the chelator-antibody molar ratio (average number of chelators per antibody). It is typically assessed using a radiometric isotopic dilution assay [20], spectrometry based UV/Vis absorption [21,22], or matrix-assisted laser desorption/ionization mass spectrometry (MALDI-TOF MS/MS) [23]. Furthermore, during a new chelator development, stability of chelator on antibody must be validated. Finally, the chelator-radionuclide complex must have sufficient thermodynamic stability and kinetic inertia to withstand possible transchelation and transmetallation in vivo [17].

### 2.1. Non-Site-Specific Bioconjugation

**Figure 3:**
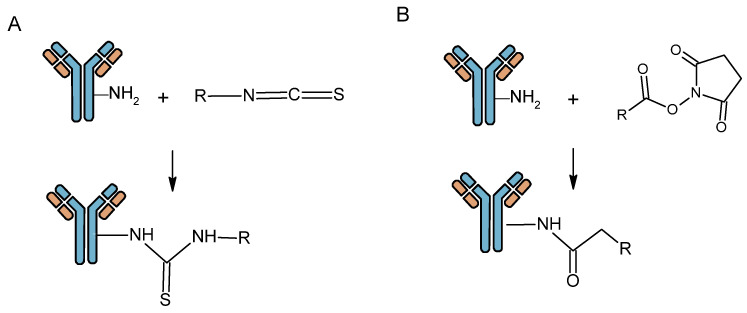
Antibody bioconjugation with *N*-hydroxysuccinimide ester (**A**) and isothiocyanate functions (**B**).

Conjugation is mainly performed on amine groups through a covalent bond on the vector to allow subsequent molecule radiolabeling. This is very advantageous as amines are very abundant on the antibody surface (cysteine or lysine residues). Several functionalities can react with amines, the most commonly used being N-hydroxysuccinimide esters (NHS) and isothiocyanates (NCS) [24] (Figure 3).

One of the main drawbacks of this method is that the probe can be conjugated to amino acid residues present on the CDR of the antibody, particularly if the number of chelating agent per antibody is important, which can lead to stoichiometric congestion and consequently to a reduction in affinity and immunoreactivity towards the antigen [19]. A second problem is the lack of control over the number of chelating agents grafted onto an antibody (chelator-antibody ratio), which leads to a lack of homogeneity among the immunoconjugates, which is a limitation particularly in the use of radiolabeled antibodies in radioimmunotherapy. More precisely, while the average number of chelators per antibody can be determined, the number of chelator molecules coupled per antibody molecule is heterogeneous and can be represented by the Poisson distribution. Impaired affinity and conjugate heterogeneity, both in terms of chelator/antibody ratio and conjugation site, can lead to less efficient in vivo behavior of the conjugate with reduced immunoreactivity. This limitation opened the door to the development of site-specific bioconjugation, which we will develop in the next section. 

**Figure 4:**
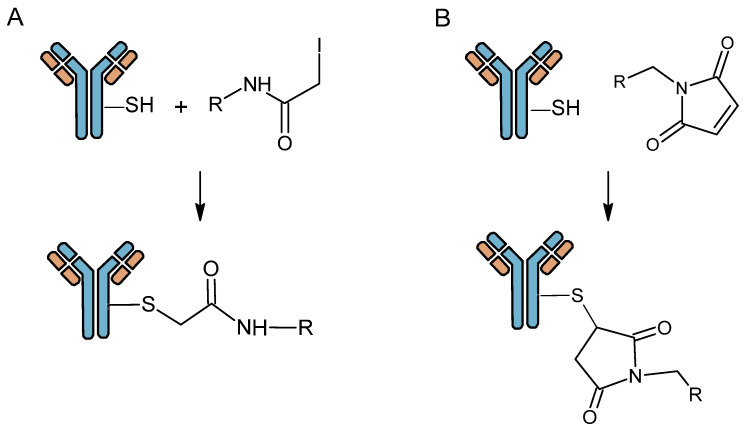
Antibody bioconjugation with iodoacetyl ester (**A**) and maleimide functions (**B**).

A second, less common method is non-site-specific cysteine conjugation. Cysteine is less common than lysine residues. A monoclonal antibody naturally contains several dozens cysteines, potential binding sites via the thiol function, which are released after a reduction step. So, it is relatively unlikely that the pharmacokinetics of proteins will be altered by limiting the number of BFCAs grafted. Nevertheless, the oxidized state of the cysteines is important for the formation of disulphide bridges that help to maintain the three-dimensional structure of the antibody. To prevent the protein from losing its structure and therefore its biological activity, gentle reduction techniques have been developed to control the number of cysteines reduced. This method focuses on the sulfhydryl (-SH) or thiol groups present on the cysteines of the antibody disulfide bridges [25]. The moieties capable of binding to thiols are generally maleimides and haloacetyls (Figure 4). As these are at the level of the disulfide bridges and variable parts of the antibody, reduction of cysteines can lead to a reduction in immunoreactivity and modify distribution profiles of radiolabeled mAb [26]. 

### 2.2. Site-Specific Bioconjugation

Efficient site-specific bioconjugation strategies have been developed using various methods to achieve chemoselectivity [27,28,29,30]. Precise control of the number and location of chelators on the antibody is expected to lead to more efficient immunoconjugates. Much progress has been made in recent years in the development of site-specific conjugates and click chemistry, as evidenced by numerous articles and reviews in the literatures [27,28,29,31,32].

#### 2.2.1. Cys Engineering

To overcome the reduction required for non-site specific cysteine technic, a method was developed to add a new cysteine to create a new labeling site [19]. Cysteine conjugation allows the reversible attachment of a cysteine-maleimide moiety in a relatively simple and straightforward manner without the need for specific reagents or enzymes. Although there is potential instability in vivo depending on the location of the modified Cys residue, this is a widely used method for site-specific radiolabeling of mAb. New methods allow irreversible Cys bioconjugation [33]. Nevertheless, the reaction is possibly reversible in cysteine-rich environments, and experience shows that such conjugates are prone to being unstable in vivo [34]. However, this method requires modified antibody vectors and may raise issues with regard to regulatory aspects and immunogenicity.

#### 2.2.2. Enzymatic and Chemoenzymatic Methods

Conjugation by chemoenzymatic modification methods was studied using several techniques: glycan modification with multi-step transformations using α-2,6-sialyltransferase, β-1,4-galactosidase and mutant β-1,4-galactosyltransferase, and sortase A-mediated conjugation [35,36]. Enzymatic methods can also allow site-specific conjugation of the chelating agent to the antibody. Enzymatic methods can be used to modify the degree of glycan functionality on the Fc fragment of a whole antibody. This modification allows the chelating agent to be specifically grafted onto the Fc fragment, thereby avoiding clogging of the Fab fragment. This preserves the immunoreactivity and affinity of the antibody. Enzyme pairs are involved: the β-1,4-galactosidase enzyme/β-1,4-galactosyltransferase enzyme (Y289L) or the PNGaseF/translutaminase couple. Several examples have been found in the literature, including antibody radiolabeling with DFO-^89^Zr and CPTA-^64^Cu [19]. A monovalent antibody fragment targeting EGFR was developed with a sequence recognized by sortase A. Two different metal chelators, H3DFOQSqOEt and MeCOSarNHS, for ^89^Zr and ^64^Cu respectively, were modified with an N-terminal glycine to allow binding with the antibody fragment by the enzyme [37]. This type of conjugation is often used to functionalize nanobodies as it reduces the impact on the affinity and immunoreactivity of the nanobody. This method produces more homogeneous immunoconjugates. A recent study demonstrated that the ^89^Zr-DFO-trastuzumab developed using this chemoenzymatic strategy outperformed its counterpart developed using the random conjugation method because the site-specifically modified ^89^Zr-DFO-trastuzumab showed increased immunoreactivity and stability in immunoPET imaging studies [38].

#### 2.2.3. Peptide Tags

In order to achieve site-specific modifications, antibodies can be produced with short peptide tags using standard protein engineering and recombinant expression protocols. Peptide tags can be used as recognition sites for enzymes to facilitate the creation of specifically modified conjugates. Peptide tags can also act as chelators themselves [32]. For example, the incorporation of the His6 tag is typically at the C-terminus of the target protein, although N-terminal incorporation is also possible. Recent examples include an anti-HER2 sdAb [39], an αvβ6 integrin-directed diabody [40]. Modifications using this technique generate payload-coupled antibody conjugates with high selectivity and stability, although the potential immunogenicity of the introduced peptide tags is currently unknown.

#### 2.2.4. Click Chemistry

More recently, a functionalization approach based on “click chemistry” has emerged [41]: these reactions are bioorthogonal, rapid, stereospecific, and enable very good yields to be obtained under optimal conditions for antibodies [42]. Click chemistry reactions use chemical functions that are not found naturally in the biological environment, giving a directed functionalization reaction. Click chemistry has been designed for different applications [39]: radiolabeling reactions with prosthetic groups, creation of new chelation architectures, site-specific bioconjugation, in vivo pre-targeting. Among the various reactions, the Cu(I)-catalyzed cycloaddition between azides and alkynes (CuAAC) is widely used in the development of radiopharmaceuticals. The first studies began with Fluorine 18 (^18^F). Marik and Sutcliffe first described the preparation of ^18^F-labeled peptides using the CuAAC reaction in 2006 [43]. Therefore, for the design and synthesis of radiotracers for molecular imaging, ^18^F-CuAAC is a very attractive strategy and several radiotracers have been developed [44,45,46]. 

A recent study showed that it was possible to radiolabel antibodies by CuAAC click reaction using a chelator (PCB-TE2A-alkyne) [47]. Tumor models are used to evaluate the feasibility of targeting tumors with radiolabeled antibodies. Results show that the ^64^Cu-PCB-TE2A-Trastuzumab conjugate has excellent in vivo stability and binding affinity to HER2-positive cells. Bioorthogonal chemistry involving azide-alkyne cycloaddition (SPAAC) may therefore prove to be an interesting alternative [48]. The SPAAC reaction, which stands for strain-promoted azide-alkyne cycloaddition, involves the cycloaddition of azides with alkynes of the cyclooctyne or dibenzocyclooctyne (DBCO) type. DBCO is readily available in industry, avoiding the necessity for complex synthesis.

Click chemistry offers two main advantages. Click reactions are generally fast and highly selective, allowing efficient production. A second advantage is that click reactions occur under mild conditions, such as room temperature and aqueous environments, minimizing the need for stringent reaction conditions. However, one limitation is that some click chemistry reactions require metal catalysts, such as copper, which can pose compatibility problems in biological systems and competition with radiometals. Especially, the radioactive copper ions are used with nanomolar or picomolar proportions, whereas the copper catalyst is introduced in microgram to milligram quantities, overflowing all coordination sites in the BFCA. In addition, the commercial availability of the necessary reagents can still be a challenge for researchers.

## 3. ImmunoPET Radiometals: Zirconium 89 and Copper 64

As there have ideal properties for radiolabeled antibodies, ^89^Zr and ^64^Cu are used for immunoPET. Table 1 shows the main characteristics of these 2 radiometals.

### 3.1. Zirconium 89

#### 3.1.1. Nuclear Properties and Production

**Figure 5:**
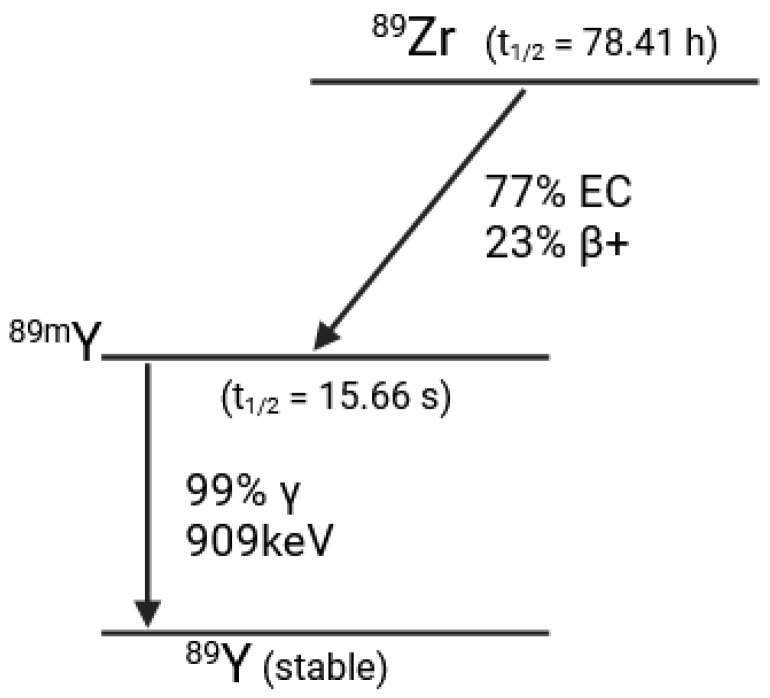
Zirconium 89 decay diagram.

Of the various zirconium isotopes, ^89^Zr has attracted the most attention for radiopharmaceutical development due to its favorable nuclear decay characteristics, which make it useful for PET. ^89^Zr is neutron deficient, with 49 neutrons and 40 protons, and has a physical half-life of 78.4 h. Its decay pattern split between electron capture (77%) and positron emission (23%) (Figure 5).

Both decay pathways lead to the formation of metastable ^89^Y, which de-excites with the emission of a 909 keV gamma ray. ^89^Zr emits two important types of radiation: the 511 keV radiation resulting from the annihilation of the positron, and the 909 keV radiation from the de-excitation of ^99m^Y.

^89^Zr is produced by a cyclotron using the reactions ^89^Y(p,n)^89^Zr and ^89^Y(d,2n)^89^Zr. ^89^Zr can be obtained by proton bombardment on ^89^Y, the isotopic abundance of the target is 100%, which makes production less costly [49]. It has been shown that the most favorable proton energy for bombarding the yttrium target is 14 MeV [50]. Beyond 13 MeV, an isotopic impurity is produced, ^88^Zr, with a half-life of 83.4 days, whose daughter isotope is ^88^Y with a half-life of 106 days, which can be problematic in terms of dosimetry and waste management. The second possible reaction to obtain ^89^Zr is to use a deuteron beam with a minimum energy of 5.6 MeV on an ^89^Y target. This method limits contamination by ^88^Zr, as the threshold energy for obtaining this radioisotope is 15.5 MeV [51].

#### 3.1.2. Zirconium Complexation Properties

Zirconium belongs to the family of transition metals [52]. There are various oxidation states of zirconium, Zr(II), Zr(III) and Zr(IV), but it is generally found as the +IV ion in aqueous solution [49]. It is generally available in solution as either zirconium-89 oxalate (^89^Zr(ox)_2_) or zirconium-89 chloride (^89^ZrCl4), thus influencing the chemistry for the development of effective immunoPET agents [53].

The cation Zr^4+^ is an ion characterized by a high charge, forming complexes with a high number of coordination bonds (up to 8 bonds), preferably with anionic oxygen. So, Zr^4+^ requires a polydentate ligand with 6 to 8 donor atoms. Currently, experimental results show that due to its high charge and small radius, Zr(IV) exists as a monomeric and polynuclear species in aqueous solution at low pH. Indeed, the high charge density on the Zr^4+^ favors the deprotonation of H_2_O molecules to form coordinated hydroxide which acts as a ligand. However, when solutions of the tetramer are acidified, monomeric species of Zr^4+^ are formed by protonation of the bridging hydroxo groups. The nature and abundance of these species can change as a function of pH, e.g., increasing pH favors the formation and precipitation of zirconium hydroxide species. This makes it more difficult to determine accurate stability constants with chelating agents [51].

#### 3.1.3. Zirconium Metabolism

The behavior of free Zr^4+^ in biological systems has been studied in order to assess the safety of using ^89^Zr as a PET tracer and to better understand the fate of radiopharmaceutical in the body. This is particularly important for zirconium because of its high bone affinity [54]. In its basal state, the body contains small amounts of zirconium in fat, liver and gallbladder, and traces in the brain. Excretion of exogenous zirconium from the diet involves the hepatobiliary system, resulting in fecal rather than urinary excretion. There are still questions about the fate of zirconium within the body. One study showed that Zr-chloride accumulates in the liver, suggesting that it is in colloidal form, while another study using a different preparation method for Zr-chloride showed no accumulation in soft tissues. Zr-DFO is eliminated very rapidly by the kidneys and can be excreted in the urine within minutes [52]. Zirconium has a strong affinity for bone, so when it is not tightly bound to a chelate or is transchelated, it preferentially accumulates in this tissue, which has been observed in rats and mice. This behavior is particularly important to consider in the clinic, as its accumulation in bone can be problematic in practice. In addition, a comparison of bone absorption from different zirconium species shows that bone absorption is highest when Zr-chloride is injected, followed by Zr-oxalate, then Zr-citrate, and finally Zr-DFO with significantly less bone absorption. It should be noted that early clinical trials with ^89^Zr do not demonstrate the extent of bone absorption shown in the preclinical images [54].

#### 3.1.4. Zirconium 89 Chelation

We will particularly focus on chelating agents that may be useful for chelating ^89^Zr to enable radiolabeling antibody. Some chelators are already used clinically: DFO derivatives and hydroxypyridinones [55], but research on chelators is still very dynamic in order to increase in vivo stability.

##### Non-Hydroxamate Chelating Agents

**Figure 6:**
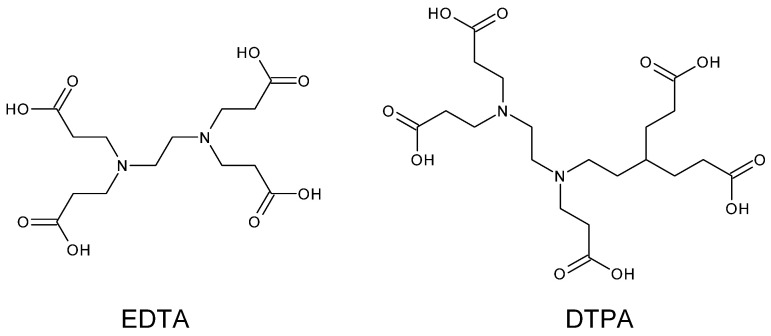
Non-hydroxamate chelators: ethylenediaminetetraacetic acid (EDTA) or diethylenetriaminepentaacetic acid (DTPA).

In early studies of ^89^Zr in PET imaging, common metal chelators such as ethylenediaminetetraacetic acid (EDTA) or diethylenetriaminepentaacetic acid (DTPA) were used (Figure 6). DTPA is able of saturating the zirconium coordination sphere. Nevertheless, DTPA-^89^Zr provides a reduced stability compared to desferrioxamine (DFO) [56]. 

**Figure 7:**
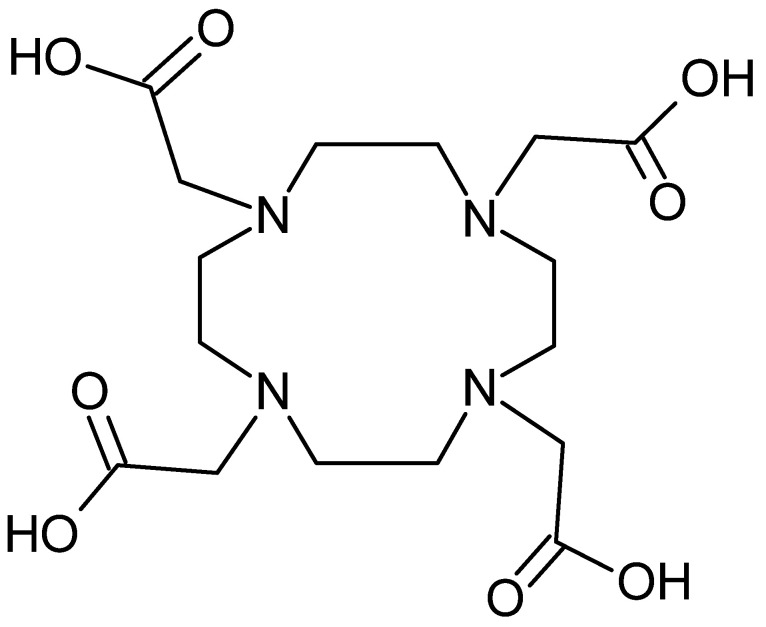
DOTA structure.

Among non-hydroxamate ligands, it is important to mention macrocyclic ligands. In PET imaging, the chelating agent DOTA is considered the gold standard for ^64^Cu and ^68^Ga. It is characterized by 4 oxygens and acetate groups that act as strong electron donors, which is not relevant for Zr^4+^ chelation (Figure 7). Pandya et al. have shown that tetraazamacrocycles such as DOTA, and its derivatives (DOTAM, DOTP) are capable of forming very stable complexes with ^89^Zr [53]. 

Initial attempts with ^89^Zr in oxalate form were unsuccessful, but the use of the chloride or acetylacetonate form of ^89^Zr was successful. However, the use of these chelating agents requires working at a high temperature (90 °C) for radiolabeling, which makes it impossible to apply them to a mAb. In a recent paper, the same group evaluated small macrocyclic chelatants such as 2,2′,2″-(1,4,7-triazacyclononane-1,4,7-triyl)triacetic acid (NOTA) and 3,6,9,15-Tetraazabicyclo [9.3.1]pentadeca-1(15),11,13-triene-3,6,9-triacetic acid (PCTA) to overcome the high temperature barrier. In fact, NOTA and PCTA could be radiolabeled under mild conditions (37 °C), and the zirconium chloride radiolabeled conjugate showed good in vitro and in vivo characteristics [53].

##### Hydroxamate Chelating Agents: Desferrioxamine and Its Derivatives

**Figure 8:**
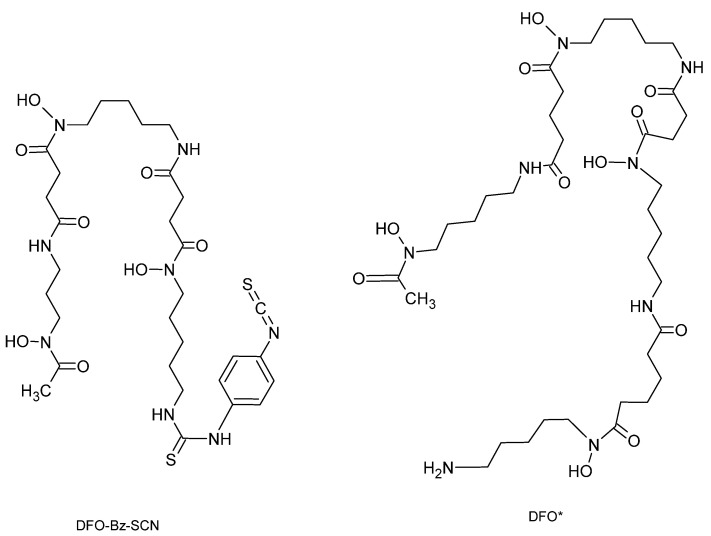
Structure of the ligand Desferoxamine-Bz-SCN (DFO-Bz-SCN) and Desferoxamine (DFO*).

DFO is also known as deferoxamine, desferrioxamine B, desferoxamine B, MPO-B, DFOA, DFB or Desferal, and is the most widely used chelator for ^89^Zr-immunoPET (Figure 8). DFO is hexadentate and has superior chelating properties for zirconium compared to non-hydroxamate chelators such as DTPA. The most likely explanation lies in the Pearson concept (HSAB theory). DTPA coordinates via three nitrogen atoms and five oxygen atoms, whereas DFO offers six oxygen donors, 3 anionic and 3 neutral.

For the introduction of DFO into two different mAb (E48 and 323/A3), Meijs et al. used a two-step procedure [56]. In the first step, maleimide groups were introduced into mAb by reaction of lysines and DFO was converted to its S-acetyl protected thiol derivative (DFO-Sac) by reaction with N-succinimidyl-S-acetylthioacetate. The bioconjugates were radiolabeled with ^89^Zr with a specific activity of 185 MBq/mg and a yield of 95%. Biodistribution studies in tumor-bearing mice showed a high tumor uptake of the conjugates, but also a high background accumulation of ^89^Zr in other tissues. This is thought to be due to poor in vivo stability, releasing free zirconium from the chelator, which has an affinity for bone. Moreover, Verel et al. also showed that linker instability could release DFO-^89^Zr complex [57]. To improve stability and facilitate bio-conjugation, DFO derivatives have been developed. A DFO derivative grafted with an activated ester (DFO-N-Suc-TFP) was synthesized [58] with a multistep protocol requiring chelator protection with Fe^3+^. Removal of the iron after conjugation is necessary but requires working under acidic conditions, which can have deleterious effects on certain biomolecules.

**Figure 9:**
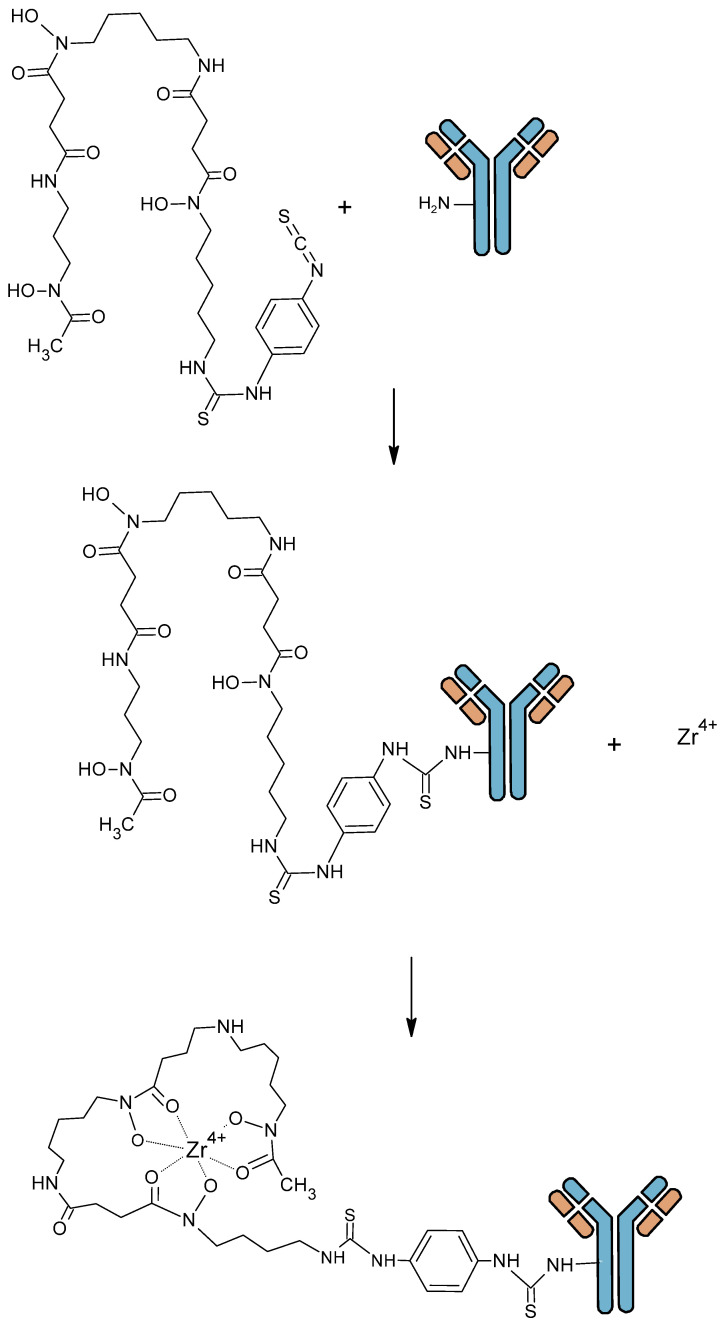
Zirconium 89 antibody radiolabeling with Vosjan et al. procedure [58].

Vosjan et al. have developed a second derivative, which is currently widely used in clinical indications, with an isothiocyanate function (DFO-pPhe-NCS). This method gave good results: optimized conjugation with high radiochemical purity, conservation of immunoreactivity and antibody stability [58]. The development of this new derivative of desferrioxamine B has enabled simpler and faster preparation of mAb conjugates. This process involves the initial coupling of Df-Bz-NCS to the lysine-NH_2_ groups of an mAb under alkaline conditions (pH 8.9–9.1) followed by radiolabeling with ^89^Zr in oxalate form (Figure 9). This method is currently the most widely used in the literature. Compared to DFO-N-Suc, in vitro stability of both conjugates was similar, with less than 4.7% release after 7 days storage in human serum. Biodistribution and PET imaging data for the two conjugates showed similar accumulation of ^89^Zr in organs and tissues.

More recently, a second team, Sharma et al. [23], worked on optimizing radiolabeling with this derivative. This new protocol allowed yields of over 98% (for a molar ratio of 5) with an immunoreactive fraction of over 92%, while saving time (60 min for conjugation and 60 min for radiolabeling at 37 °C). For comparison, at a molar ratio of 5, Sharma et al. found the number of chelates per antibody to be 1.4 ± 0.5 (by MALDI-TOF), whereas at a molar ratio of 1.5, Vosjan et al. [58] obtained a number of chelates per mAb between 0.3 and 0.9. This may explain the difference in yield between these two protocols. It should also be noted that Sharma et al. chose to work under metal-free conditions, i.e., conditions that minimize possible metal contamination [23]. They also investigated the effect of the number of chelators on affinity and found that a high molar ratio of chelator leads to a decrease in affinity.

As mentioned above, it is also possible to functionalize the antibody with cysteine functions, allowing site-specific conjugation. Several chelators have been developed: DFO derivative bromoacetamido-desferrioxamine (DFO-Bac) with a thiol function, iodoacetamido-desferrioxamine (DFO-Iac), and maleimidocyclohexyl-desferrioxamine (DFO-Chx-Mal) [52]. The first two chelators are conjugated to the antibody via a nucleophilic substitution reaction on a non-active site, whereas DFO-Chx-Mal requires a Michael reaction. The antibodies obtained showed good stability after radiolabeling. However, despite site-specific conjugation, no improvement in immunoreactivity was reported [59].

More recently, an ethylenediamine platinum(II) moiety was attached to the DFO via a linker to create DFO-Lx. Using this chelating agent, trastuzumab was radiolabeled with ^89^Zr by Sijbrandi et al. [60]. This radiolabeled antibody was compared with its ^89^Zr-DFO-trastuzumab counterpart. No major differences in pharmacokinetics were observed, except for a greater hepatic accumulation of the Lx conjugate.

From DFO, a second derivative was synthesized, DFO* (Figure 8), which is distinguished by the incorporation of an extra hydroxylamine function by a coupling reaction between the DFO salt and a carboxylate function [61]. DFO* was studied with radiolabeled trastuzumab. DFO*-mAb showed superior properties in vitro and in vivo, including a biodistribution less intense in the bones than DFO-trastuzumab at 144h post-injection [62]. Furthermore, DFO*-trastuzumab enabled more accurate detection of bone metastases, illustrating the importance of stable chelation [63]. It should be noted that trastuzumab-DFO*-^89^Zr is being studied in a clinical trial (NCT05955833).

A squaramide ester derivative of DFO (DFO-Sq) was proposed by Donnelly et al. for conjugation to trastuzumab, similar to the DFO* approach [64]. When injected into SK-OV-3 xenograft-bearing mice, the new conjugate improved PET imaging and resulted in a higher tumor-to-bone ratio. Other DFO derivatives have been developed, such as DFO-Cyclo* [65].

Some of the chelators derived from DFO are already used clinically: Fe-DFO-N-suc-TFP ester, p-NCS-Bz-DFO, Mal-DFO, p-NCS-Bz-DFO*, Mal-DFO*, Sq-DFO* [55].

##### HOPO-Based Ligands

**Figure 10:**
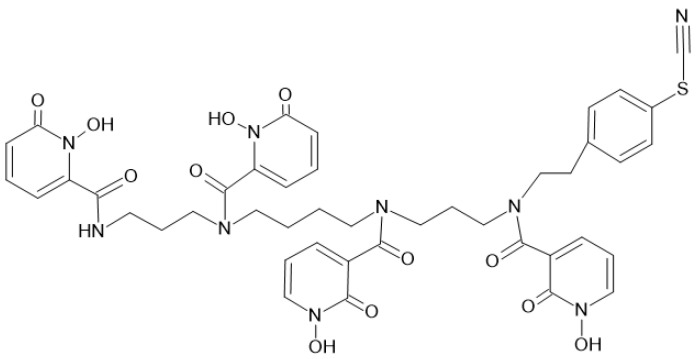
Structure of p-SCNBn-HOPO.

Historically, White et al., for the chelation of plutonium (IV), investigated octadentate hydroxypyridinonate (HOPO) ligand. HOPO are another family of chelating agents suitable for zirconium. They are characterized by a high pKa, which gives them the ability to ionize at neutral and acidic pH. A bifunctional version of HOPO with a benzyl NCS group have been developed to bioconjugate trastuzumab. However, in vivo studies in BT474 xenograft-bearing mice confirmed the superiority of the new conjugate, particularly with respect to bone uptake [64]. The chelator 1,2 HOPO allows to complex ^89^Zr, developed by Deri et al. showed a superiority to DFO [66]: complex was eliminated by renal excretion and bone fixation of free zirconium was reduced. These results encouraged the same team to work on a bifunctionalized version, p-SCNBn-HOPO (Figure 10), with good tumor and low bone uptake [67]. This chelator has been used clinically. 

The 2,3HOPO was also studied, but unfortunately these bioconjugates did not offer any reduction in bone accumulation compared to DFO [59]. Other ligands have shown excellent in vivo chelating properties, with rapid complex elimination and no evidence of transmetallation, such as the tetrapod ligand 3-hydroxy-4-pyridinone (THPN). Based on four 3-hydroxy-4-pyridinone (3,4-HOPO) coordinating groups, in an attempt to fully satisfy the octadentate coordination sphere preferred by the ^89^Zr^4+^ ion [68].

**Figure 11:**
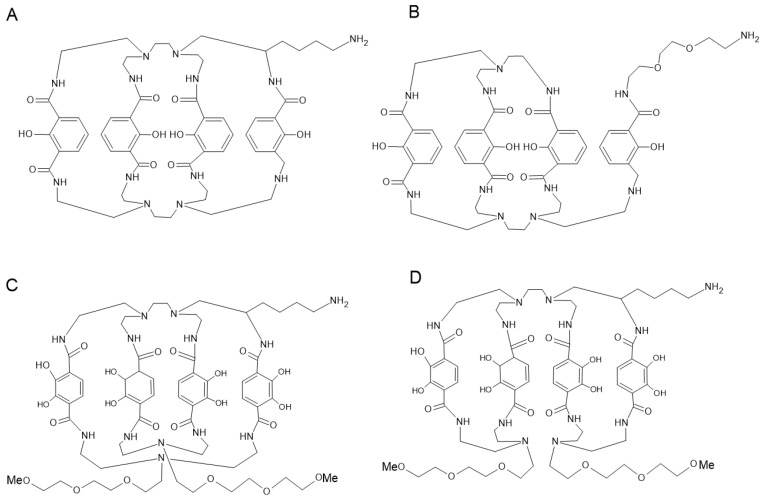
Structure of IAM-1 (**A**), IAM-2 (**B**), TAM-1 (**C**), TAM-2 (**D**).

Di-macrocyclic hydroxyisophthalamide ligands (IAM-1, IAM-2) and di-macrocyclic terephthaphthamide ligands (TAM-1, TAM-2) have been developed for the complexation of ^89^Zr (Figure 11).

These octadentate ligands can be radiolabeled quantitatively and show good in vitro stability. The team led by Pandya et al. focused on the molecules TAM-1and TAM-2 and demonstrated, using in vitro studies and PET imaging, the high stability of this type of complex with bone fixation comparable to that of DFO [69].

##### Other Hydroxamate Ligands Not Derived from DFO

An initial study was carried out using fusarinin C (FSC), derived from natural siderophores, to perform radiolabeling with ^89^Zr. In addition, FSC contains three primary amines that allow more functionalization, for example to increase its denticity. Derivatives such as triacetylfusarinin C (TAFC) have been developed from this FSC. In vitro experiments have shown greater stability to trans-chelation and transmetallation. 

### 3.2. Copper 64

#### 3.2.1. Nuclear Properties and Production

**Figure 12:**
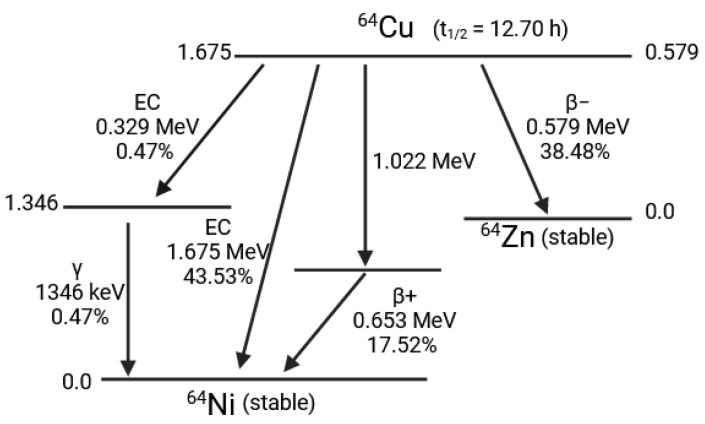
Copper 64 decay diagram.

There are 29 isotopes of copper, including ^64^Cu. ^64^Cu has 29 protons and 35 neutrons and has a physical half-life of 12.7 h. Its decay is complex, with 20% β^+^ emission (giving nickel 64), 40% β^−^ emission and 40% electron capture. The gamma emission of 511 keV is emitted after the β^+^ emission, and the high energy gamma emission (1346 keV) from electron capture is very low (0.47%) (Figure 12). These characteristics make them suitable for clinical use in PET, but also for theranostic applications.

Copper 64 is produced either in a reactor by irradiating copper 63 with neutrons (^63^Cu(n,γ)^64^Cu), or using an accelerator from the reaction ^68^Zn(p,2n)^67^Ga. However, the latter production method is not very favorable for the production of copper 64 because gallium 67 is the main product of the reaction and has a long half-life (72 h). A second accelerator method is based on the use of deuterons on a nickel 64 target. After the production of copper, there may be impurities in the solution like Zn^2+^, Ni^2+^ (from the decomposition of ^64^Cu), Co^2+^ (from target irradiation) and Fe^3+^ (from the environment). The presence of these metals can interfere with the complexation reaction with the ligand present on the vector.

We cannot describe ^64^Cu without mentioning another Cu isotope, ^67^Cu, which, with a half-life of 2.58 days, is known for its potential in both therapy and Single Photon Emission Computed Tomography (SPECT) imaging. It decays by β^−^ (Eβ-max: 562 keV) and γ (93 keV and 185 keV).

#### 3.2.2. Copper Complexation Properties

Copper is a chemical element with atomic number 29 and belongs to the family of transition metals. It can be found in solution in three oxidation states (+I, +II and +III) and is most commonly found in the +I and +II forms. These different states can modify the kinetics and stability of copper complexes [70]. In particular, Cu(I) can form relatively unstable complexes due to its saturated 3d orbital. Cu(I) forms complexes with compounds that are not very polarizable (cyanides, thiolates or nitriles) and not very soluble in water. In aqueous solution, the +II oxidation state is the most common and forms stable complexes with amines, imines or pyridines. It is important to note that the redox potential of the Cu(II)/Cu(I) pair is rather low (0.153 V/Standard Hydrogen Electrode), so this pair is easily reduced or oxidized physiologically [71].

#### 3.2.3. Copper Metabolism

Copper is absorbed from food into the digestive tract, mainly in the duodenum. This exogenous Cu(II) must be reduced to Cu(I) by reductases present in cell membrane. After reduction, copper is transported intracellularly by copper transporter 1 (Ctr1) proteins, whose expression is regulated by the level of intracellular copper. A chaperone protein, Antioxidant 1 copper chaperone (Atox1), captures the copper and delivers it to an ATPase enzyme (ATP7A), which releases the copper into systemic circulation. Blood distribution is mediated by albumin and α2-macroglobulins such as transcuprein or ceruleoplasmin. Proteins present in hepatocytes, including Atox1, bind excess copper to excrete it in bile. Copper is involved in the proper functioning of many enzymes and proteins, such as Superoxide Dismutase (SOD), which plays an antioxidant role, or protein CCO (cytochrome oxidase), which plays a role in the synthesis of ATP or ATPases. After hepatic metabolism, copper is taken up by ceruleoplasmin, an α2-glycoprotein capable of complexing 6 copper atoms. It is then called holoceruleoplasmin and enters the circulation to act as a cofactor for transferrin.

#### 3.2.4. Copper Chelation

Some copper chelators are already used clinically as ATSM (diacetyl bis(*N*-4-methylthiosemicarbazone)), DOTA, NOTA [72].

##### Acyclic Ligands

The first ligand systems used were acyclic carboxylate polyamine ligands such as EDTA, DTPA and their derivatives (Figure 6). Despite good thermodynamic stability, serum stability studies using ^67^Cu-labelled ligands showed that the complexes were not stable in human serum. This may be due to reduction of Cu(II) to Cu(I) or transchelation reactions [73]. Other acyclic ligands that have enjoyed moderate success as chelators of ^64^Cu include thiosemicarbazone ligands, in particular ATSM [74] which is generally used to study cellular hypoxia [75]. 

##### Azamacrocyclic Ligands

**Figure 13:**
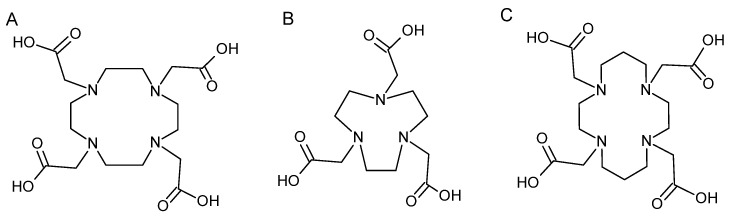
Structure of DOTA (**A**), NOTA (**B**) and TETA (**C**).

To improve in vivo ^64^Cu complexes stability, the researchers turned to tetraazamacrocyclic ligands. Since Cu^2+^ is a Lewis acid with a preference for strong donors, polyazamacrocyclic ligands have dominated its complexation. Within this family, there are tetraazamacrocyclic chelating agents such as cyclam and cyclen. The main ligands found are DOTA, NOTA, 1,4,8,11-tetraazacyclotecedecane-1,4,8,11-tetraacetic acid (TETA) and their derivatives (Figure 13).

The improved stability is undoubtedly due to a more suitable geometry with the macrocyclic ligand, which improves the kinetic inertia and thermodynamic stability of ^64^Cu complexes. However, in vivo, the radiometal is released and accumulated in the liver and kidneys of murine models [76]. 

DOTA is the chelating agent most frequently found in literature for mAb radiolabeling with ^64^Cu. DOTA is functionalized to give p-SCN-Bn-DOTA (isothiocyanate activated form) and NHS-DOTA (activated mono-ester form). DOTA is relatively poorly selective for copper, potentially leading to transmetallation. TETA ligand and its derivatives improve in vivo stability, but remain susceptible to transchelation mechanisms, particularly in liver [77]. Finally, NOTA has been shown to complex copper rapidly and in several studies to increase in vivo stability [78].

**Figure 14:**
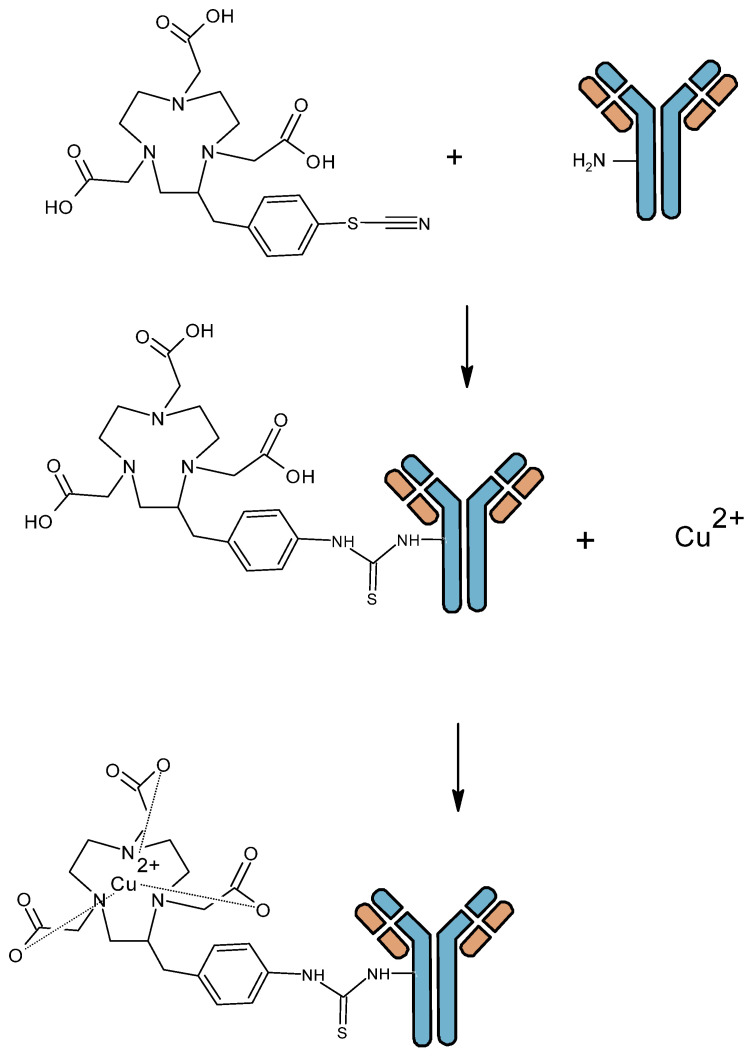
Copper 64 antibody radiolabeling with NOTA ligand.

NOTA forms a 6-coordinate complex with copper through coordination of three nitrogen atoms and three carboxyl groups of the chelator (Figure 14). In a study, trastuzumab was conjugated with SCN-Bn-NOTA to be radiolabeled with ^64^Cu. The ^64^Cu-NOTA-trastuzumab obtained a high radiolabeling yield and radiochemical purity (≥98%), verified by instant thin layer chromatography. In vitro stability of the ^64^Cu-NOTA-trastuzumab in PBS, mouse and human serum at 37 °C showed an highly stability in PBS, mouse and human serum for 48 h at 37 °C and a decrease of <3% and <2% for 24 h at 4 °C. ^64^Cu-NOTA-trastuzumab immunoreactivity was conserved (0.92 In vivo stability was demonstrated in a xenograft model of HER2-positive breast cancer (BT-474) [79]. 

To evaluate chelator/antibody ratio, UV/visible assay techniques based on the method of Brady et al. can be used for azamacrocyclic ligands [21].

##### Sarcopagin-like Ligands

**Figure 15:**
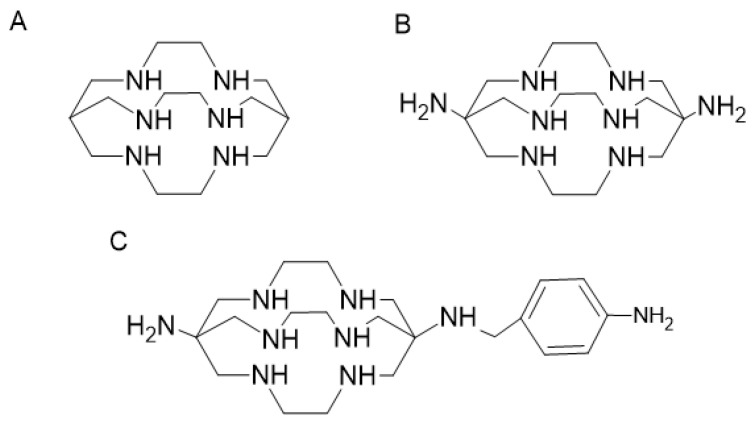
Structure of sarcophagnie (Sar) ligands: Sar (**A**), Diam-Sar (**B**), SarAr (**C**).

The hexaazamacrobicyclic or sarcophagine (Sar) ligands are another class of TETA-derived ligands that have attracted attention as potential copper chelators (Figure 15). 

These chelators were initially described by Bartolo et al. and have shown excellent thermodynamic stability and good selectivity for ^64^Cu [80]. Several studies have indicated that Sar ligands are able to form extremely stable complexes with copper with good in vivo stability [78,81,82,83,84]. However, the complexation kinetics are slower at room temperature. Bartolo et al. investigated Sar derivatives with different functions and showed a high stability of ^67^Cu-Sar complex in human plasma, with 98% of activity complexed after 7 days. Biodistribution studies were also performed with ^64^Cu-Sar, ^64^Cu-diam-Sar and ^64^Cu-SarAr in Balb/c mice. Uptake in bone, heart, stomach, spleen, muscle, lung and gastrointestinal tract was low. Hepatic clearance was observed over 30 min, indicating that ^64^Cu complex is initially stable in vivo [85].

##### Bridged Cyclam and Cyclen Ligands

**Figure 16:**
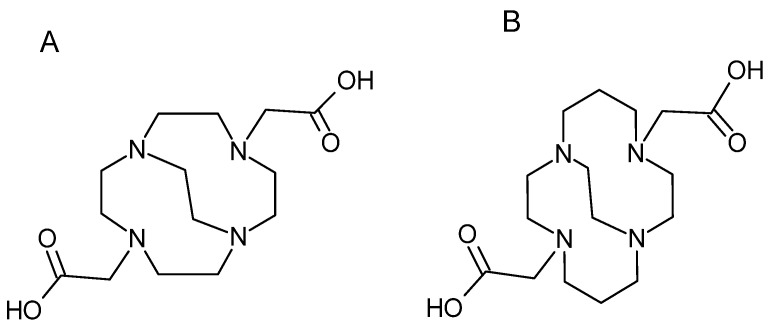
Structure of the ligands CB-DO2A (**A**) and CB-TE2A (**B**).

More recently, the cyclam and cyclen ligands and their derivatives have attracted attention for complexing copper. They are composed of an ethyl bridge that links the amine functions N1 and N4, which is also called “cross-bridged” (Figure 16).

These macrobicyclic chelating agents were synthesized to chelate metal cations such as Li^+^, Cu^2+^ and Zn^2+^. Among these bridged ligands, derivatives of DOTA and TETA have been developed, which are B-DO2A and CB-TE2A respectively. These ligands are obtained by introducing an ethylene group between two opposite nitrogen atoms, which enabled complete envelopment of a six-coordinate Cu(II). The addition of this function increased the thermodynamic stability and inertia of the copper complexes, particularly with TE2A [86]. In addition, Cu-CB-TE2A showed excellent resistance to the reduction of copper from its Cu(II) form to the Cu(I) form [87]. However, due to severe functionalization conditions, including heating to 95 °C, the use of CB-TE2A for mAb labeling is limited [86].

It should be noted that a more recent derivative, CB-TE1A1P [(1,4,8,11-tetraazacyclotetradecane-1-(methanephosphonic acid)-8 (methane carboxylic acid) can chelate ^64^Cu at room temperature. Meanwhile, the TE1A1P complex with copper shows a low in vivo stability. CB-TE1K1P, was developed for copper-based radiopharmaceuticals, and this chelator can be labeled with ^64^Cu under mild conditions in high specific activity and an improved in vivo stability [88].

## 4. Applications

Once the mAb has been radiolabeled, its characteristics need to be verified. Firstly, to determine radiochemical purity and antibody integrity, quality controls of radiolabeled antibodies are usually performed by HPLC (size exclusion chromatography) or SDS-PAGE. TLC is also used to assess radiochemical purity by molecular weight separation. Moreover, binding assays must be developed to evaluate immunoreactivity and affinity of the radioimmunoconjugate. These tests can be performed by binding cell studies [89], or by surface plasmon analysis [23]. In addition, if clinical use is envisaged, production must follow Good Manufacturing Practice (GMP) guidelines, in a clean and controlled environment, respecting a reproducible and validated manufacturing process and well-defined release criteria [17].

Before a clinical application, preclinical studies are necessary to verify the specificity and the in vivo stability of the vector. Antibodies can bind specifically to antigens located at other sites than the target (tumor), phenomenon called “antigen sink effect” [90,91]. Furthermore, non-specific interaction could also exist in vivo. These phenomena are responsible of a target-background ratio diminution and reduced image quality. To optimize mAb biodistribution to target, cold mAb could be injected before radiolabeled mAb injection or the dose of mAb injected can be increased. Furthermore, the optimum image acquisition time must be determined [92]. Nevertheless, extrapolation of preclinical data to the clinical depends on the cross-reactivity of antibodies between murine and human antigens, and the similarity of antigen expression profiles between these two species.

In this section, we describe a number of recent studies. The major developments in the chemistry and techniques of zirconium and copper chelation are evidence of the growing interest in their application in immunoPET and of their usefulness. 

### 4.1. Preclinical Studies

Two main types of preclinical applications for immunoPET can be identified in literature. Firstly, immunoPET is the method of choice for imaging tumor and evaluating specific tumor markers, immune cells, immune checkpoints, and inflammatory processes for theranostic applications. Secondly, immunoPET imaging can be useful in the development of antibody-based drugs as it allows, by visualizing the antibody, to evaluate targeting abilities and distribution profiles. Furthermore, some studies are being carried out into the development of new chelating agents to improve radiolabeling.

#### 4.1.1. Zirconium

^89^Zr radiolabeled mAb in immunoPET are even more numerous in the literature. Several studies have turned to immunoPET as a tool for quantifying antigen expression in tumors to predict mAb efficacy or to help drug development. Various immune checkpoint targets have been evaluated, like PD-L1. To overcome the heterogeneity of certain tumors and the invasive nature of biopsy required for immunohistochemistry, Kelly et al. radiolabeled an anti-PD-L1 Ab (REGN3504) with ^89^Zr using DFO chelator to study PD-L1 expression [93]. Biodistribution study was assessed in humanized mice with human tumor xenografts and pharmacokinetics has been investigated in monkeys. More intense uptake of ^89^Zr-REGN3504 in human tumor xenografts was observed by immunoPET. Ex vivo biodistribution studies demonstrated high tumor:blood ratio. Following depletive treatment of PD-L1-positive cells, ^89^Zr-REGN3504 accurately detected a significant reduction in PD-L1-positive splenic cells. This work demonstrates the ability of ^89^Zr-REGN3504 to track PD-L1 expression and suggests that this antibody could predict and monitor response to anti-PD-1 therapy. Other studies have confirmed the value of PD1/PD-L1 mapping using immunoPET. The preclinical study of ^89^Zr-DFO-anti-PDL1 in an orthotopic lung cancer murine model established a pharmacokinetic, biodistribution and dosimetric profile for the antibody [94]. Tumor uptake is visible 24 h after injection, with optimal imaging 48 h after antibody injection. ^89^Zr-DFO-anti-PD-L1 was shown to specifically target PD-L1 in CMT167 lung tumors in an in vivo blocking study. Compared to other ^89^Zr radiolabeled mAb, the predicted dosimetry was similar or lower. Thanks to this type of preclinical studies, theranostic applications of PD1/PD-L1 or other immune checkpoint targets are now being explored in the clinic (Table 2).

Ab radiolabeling for diagnostic and mapping purposes can be performed for multiple antigens such as VEGF-R, as illustrated by Novy et al. study with ramucirumab-^89^Zr [95]. One of the limitations of ^89^Zr radiolabeling using a DFO-type chelating agent is the size of the cage and the steric hindrance it can cause. It is therefore reasonable to assume that Ab fragment radiolabeling may be affected. Gosh et al. study the radiolabeling of ^89^Zr-miltuximab and its fragments in glioblastoma [96]. Authors use glypican-1 (GPC-1) as a target of ^89^Zr-miltuximab and generating fragments of this Ab (Fab’2, Fab, and single-chain variable fragment (scFv) formats) to investigate its immunoPET potential in preclinical models. This investigation showed improved tumor uptake as well as a better tumor/background ratio with the whole antibody compared to the fragments. In this study the whole antibody format is more effective and ^89^Zr-DFO miltuximab has potential for glioblastoma diagnosis in immunoPET.

#### 4.1.2. Copper

MAb radiolabeled with ^64^Cu have a wide range of applications, starting with diagnostics. ^64^Cu-radiolabeled mAb were used in the diagnosis of multiple myeloma [97]. Anti-murine CD138 mAb were grafted withTE1PA chelate, which reduces transchelation. Radiolabeled mAb was injected into an orthotopic and ectotopic immunocompetent mouse model of multiple. Imaging with ^64^Cu-TE1PA-9E7.4 provided high-resolution images. The mAb biodistribution and dosimetry study showed a favorable concentration of radioactivity in the tumor compared with healthy tissue. 

In addition to diagnostic applications, theranostic applications are also possible. As presented in the previous section, ^64^Cu, a β^+^ emitter, has a β^−^ emission which offers a possible therapeutic application. So, the anti-tumor efficacy of different activities of ^64^Cu-TE1PA-9E7.4 were studied and have shown to have an anti-tumor effect in certain doses, with a significant improvement in survival in several cohorts [97]. This anti-tumor effect demonstrates the theranostic characteristic of ^64^Cu itself.

A growing number of preclinical studies have focused on the therapeutic properties of copper, such as the study by Milot et al. In a first study, they verified the diagnostic potential of copper radiolabeled PSMA in prostate cancer [98], then, they investigated its therapeutic efficacy [99]. Overall survival was prolonged with ^64^Cu-DOTHA2-PSMA compared to control. Mice showed no signs of radiation exposure, but developed non-pathological fibrosis.

In addition to various theranostic applications of ^64^Cu-radiolabeled mAb, Yoshii et al. showed that ^64^Cu-PCTA-cetuximab can be a therapeutic and surgical tool in pancreatic cancer [100]. First, they observed a therapeutic effect of radiolabeled mAb (by intraperitoneal administration) with an increase of survival in mice, without acute toxicity. In addition, ^64^Cu-PCTA-cetuximab has demonstrated its imaging potential, allowing PET-guided microsurgery of disseminated tumor. These results demonstrate that the same radiopharmaceutical can be used to both locate tumor lesions and guide ablative surgery, and to confirm the absence of tumor remnants after surgery.

Some studies have investigated mAb prediction and monitoring potential in immunoPET. The team of Kristensen et al. proposed a study of Ab fragment targeting CD8, conjugated to NOTA and radiolabeled with ^64^Cu [101]. They investigate radiolabeled mAb capacity to predict immunotherapy response in CT26 tumor-bearing mice. They identified responders and non-responders in tumors treated with a combination of radiotherapy and CTLA-4 inhibition. Compared to non-responders, responder mice showed an increase in the ratio of ^64^Cu-NOTA-CD8a in the tumor to the heart. These results suggest that ^64^Cu-NOTA-CD8a could be used to predict response and monitor patients during immunotherapy protocols. A second study examined the ^64^Cu-NOTA-pertuzumab F(ab′)2 in trastuzumab response monitoring by PET/CT [102]. Study shows that ^64^Cu-Ab imaging can detect changes in HER2 expression in response to trastuzumab.

### 4.2. Clinical Studies

Many clinical applications are available in the literature. For the purposes of this review, we have focused on recent publications from the last 3 years from PubMED, as well as ongoing clinical trials from the ClinicalTrials.gov website (Table 2).

**Table 2:** Review of recent publications and ongoing clinical trial from the last 3 years.

Radionuclide	Antibody	Target	Indication	References
**^89^Zr**	Girentuximab	CAIX	Metastatic clear-cell renal cell carcinoma	[103]
	Daratumumab	CD38	Multiple myeloma	[104]
	CED88004S	CD8	Advanced or metastatic solid cancer	[105]
	AMG 757	DLL3	small-cell/neuroendocrine cancer	[106]
	Panitumumab	EGFR	Head and neck squamous cell carcinoma	[107]
	Trastuzumab	HER2	Metastatic oesophago-gastric cancer	[108]
	Trastuzumab	HER2	Metastatic breast cancer patients	[109]
	Trastuzumab	HER2	Metastatic esophagogastric cancer	[110]
	Pembrolizumab	PD-1	Metastatic melanoma and non-small cell lung cancer	[111]
	KN035	PD-L1	Primary and metastatic PD-L1 positive tumors	[112]
	BI 754091/BI 754111	PD-1/LAG3	Non-small cell lung cancer, head and neck squamous cell carcinomas	[113]
	IAB2M	PSMA	Prostate cancer	[114]
	Crefmirlimab Berdoxam	CD8	Advanced or metastatic melanoma, merkel cell carcinoma, renal cell carcinoma, or non-small cell lung cancer	Clinical trial—NCT05013099
	Crefmirlimab Berdoxa	CD8	Melanoma	Clinical trial—NCT05279027
	Anti-CD147	CD147	Advanced solid tumors: non-small cell lung cancer (NSCLC); Gastric adenocarcinoma; Colorectal adenocarcinoma …	Clinical trial—NCT04841421
	CB307	CD137; PSMA; and human serum albumin (HSA)	Solid tumor: PSMA+ and PSMA− Tumour Lesions	Clinical trial—NCT05836623
**^64^Cu**	αCD19	CD19	Lymphoma	[115]
	Daratumumab	CD38	Multiple myeloma	[116]
	M5A	Carcinoembryonic Antigen (CEA)	Rectal cancer, medullary thyroid cancer	[117]
	GD2-antibody	Disialoganglioside GD2	Neuroblastoma, Osteosarcoma, Ewing’s sarcoma	[118]
	Trastuzumab	HER2	Breast cancer	[119]
	Trastuzumab	HER2	Breast cancer	[120]
	Trastuzumab	HER2	Gastric tumor	[121]
	M5A	CEA	CEA positive cancers: gastrointestinal, lung, medullary thyroid and breast cancers	Clinical trial—NCT02293954
	Trastuzumab	HER2	Metastatic HER2 Positive Breast Cancer	Clinical trial—NCT02226276

#### 4.2.1. Zirconium

The first clinical trial using a radiolabeled mAb to ^89^Zr dates back to 2006, in primary carcinoma of the head and neck by the team of Börjesson et al. [122]. This first study demonstrated the feasibility and safety of radiolabeled mAb to ^89^Zr in diagnosis [120]. More recently, a the study of radiolabeled daratumumab in multiple myeloma diagnosis, which makes it possible to localize and quantify the expression of the disease [104].

However, in addition to diagnostic applications, an increasing number of studies have focused on monitoring patient response during treatment. The study by Kok et al. looked at the potential of ^89^Zr-pembrolizumab for monitoring patients treated with anti-PD-1 for metastatic melanoma or non-small cell lung cancer [111]. This study began by optimizing the protocol, they injected radiolabeled antibody with free antibody to optimize the tracer’s biodistribution. and an ideal acquisition at 7 days post-injection. Patients were treated with different immune-checkpoint inhibitor (ICI) (pembrolizumab; nivolumab; pembrolizumab and nivolumab combination; ipilimumab-nivolumab) and with radiotherapy for patients with brain metastases. At the end of treatment, response was assessed using RECIST v1.1 criteria and patients were imaged using radiolabeled antibody. Tumor uptake of ^89^Zr-pembrolizumab correlated with tumor response to treatment, progression-free and overall survival. 

A study looked at the assessment of CD8+ T lymphocyte biodistribution by PET imaging in cancer patients pre- and post-therapy with checkpoint inhibitors. Authors showed that the presence of CD8+ T cells in tumor lesions imaged by the Ab, prior to ICI, could be predictive of overall survival. This criterion highlights the potential of CD8 imaging as a predictive biomarker for personalizing patient treatment. Nevertheless, this study raises a limitation in the application of immunoPET: the choice of target. Despite its great potential, the target is questionable because it is difficult to distinguish between inflammatory and tumor phenomena [105].

In a clinical trial, ^89^Zr-radiolabeled trastuzumab was used to study HER2 expression and assess lesions in patients with metastatic oesophago-gastric cancer. ^89^Zr-Trastuzumab demonstrated the heterogeneity of tumor HER2 expression and allowed the visualization of lesions in the brain. However, this study showed that ^18^F-FDG PET detected more lesions than the radiolabeled antibody [108].

Another study using PET imaging with an Fc fusion protein of an anti-PD-L1 antibody first demonstrated the feasibility of labeling an antibody fragment using the chelating agent DFO [112]. This tracer showed significant binding at primary and metastatic tumor sites in PD-L1-positive patients. However, there was no correlation between the level of PD-L1 expression in tumor samples and antibody binding. This may be due to biopsy sampling bias or the spatial heterogeneity of the tumor, as shown in previous studies [123]. In contrast to the previous study, this study showed that PET with the radiolabeled antibody was able to detect many metastatic lesions and showed similar or even higher uptake than ^18^F-FDG PET. In addition, the antibody was able to predict and assess therapeutic response in PD-L1-positive patients, but showed less diagnostic potential in PD-L1-negative patients due to its non-specific binding.

#### 4.2.2. Copper

One of first clinical trials of a ^64^Cu-radiolabeled mAb was with trastuzumab in HER2+ breast cancer in 2012. This study was carried out in 6 patients with primary or metastatic breast cancer and showed good images at 48 h post-injection and visualization of metastases in 2 patients. This study demonstrated the feasibility and safety of using radiolabeled ^64^Cu-mAb in immunoPET [120]. A limitation of this study is that HER2 expression may change during anti-HER treatment and the authors were unable to confirm HER2 status in parallel with PET imaging [124]. Nevertheless, this study provides grounds for optimism about the value of ^64^Cu-DOTA-trastuzumab in assessing HER2 expression in breast cancer. A recent study has developed ^64^Cu-trastuzumab for the diagnosis of HER+ gastric tumors in 8 patients compared to HER2 non-expressing tumors [121]. They injected radiolabeled antibody (5 mg) with free antibody (45 mg) to optimize the tracer’s biodistribution. This study failed to demonstrate the potential of ^64^Cu radiolabeled trastuzumab with no difference between HER2 expressing and no expressing tumor. This study highlights the efforts that need to be made in the development of radiolabeled tracers in terms of antibody dose, activity and acquisition time for more relevant imaging.

Other clinical trials using ^64^Cu radiolabeling with DOTA have been reported in the literature. A study of radiolabeled daratumumab in multiple myeloma diagnosis has been conducted [116]. This study also raised an interesting question. To reduce uptake in the liver and spleen, the team injected cold antibody with the radiolabeled antibody. This co-injection has been done in other trials [121]. They found that 45 mg of cold antibody reduced liver and spleen uptake, but 95 mg reduced tumor uptake. This raises the question of the real ability of the radiolabeled antibody to be a therapeutic companion in previously treated patients. The tumor saturation sites by prior treatment may be a limit to their use. This phenomenon of cold antibody/radiolabeled antibody balance may explain the failure of the above-mentioned study [121].

Another potential indication for ^64^Cu-immunoPET is the prediction of therapeutic response in patients prior to treatment. The study by Mortimer et al. investigated the correlation between tumor uptake of ^64^Cu-radiolabeled trastuzumab and patient response to trastuzumab emtansine [119]. They conducted this study in patients with HER+ breast cancer who had not received trastuzumab in the 4 weeks prior to imaging. They showed a correlation between response to treatment as assessed by ^18^F-FDG and tumor uptake of ^64^Cu-DOTA-trastuzumab. This study demonstrates the potential of immunoPET for patient selection.

## 5. Conclusions and Future Directions

ImmunoPET is a very promising tool for non-invasive whole-body visualization of expression of tumor-associated antigens with high resolution, high specificity, high tumor uptake and a good signal to noise ratio. ImmunoPET is able to provide very informative data on the intra-tumor and intra-patient variability on molecular biomarkers expression and consecutively in phenotypic heterogeneity of the entire disease burden. It is a useful modality to predict and evaluate the response of therapeutic mAbs in order to tailor treatment for individual patients. A variety of radionuclides and mAbs have been exploited to develop immunoPET probes, which has been driven by the development and optimization of radiochemistry and conjugation strategies. However, to fully integrate immunoPET in the clinic, several hurdles still need to be overcome and appropriate radionuclide must be defined. ^89^Zr and ^64^Cu present numerous advantages for this application.

First of all, standardized and robust methods for stable conjugation to antibodies should be available to obtain clinical-grade conjugates. DFO is the most used chelator of ^89^Zr. Nevertheless, a significant amount of dechelated forms of ^89^Zr (^89^Zr-chloride, ^89^Zr-citrate, and ^89^Zr-oxalate) accumulates in the bone marrow when entered into the circulatory system due to instability of ^89^Zr-DFO chelation. Recent studies in radiochemistry are paving the way toward improving stability by changing the structure of DFO or exploiting new chelators. A decrease in bone marrow uptake is good for increasing sensitivity to lesions, as well as decreasing bone marrow toxicity for patients. Other promising alternatives are chelators with hydroxypyridinone moieties, including HOPO and 2,3-HOPO, which have higher in vitro and in vivo stability compared to DFO. Different chelators are also developed for improving stability of ^64^Cu radiolabeling even if variety of well-established chelates (DOTA, NOTA etc.) can be easily and stably labeled with ^64^Cu. In 2020, ^64^Cu-dotatate was approved by the FDA as a radioactive diagnostic agent for PET-imaging agent for SSTR-positive NETs in adult patients [125]. A limitation in the use of ^64^Cu is its hepatic biodistribution, which can lead to unwanted liver irradiation, as seen in the study by Bailly et al. comparing the same radiolabeled antibody with ^64^Cu and ^89^Zr [126]. 

Physical properties are also a critical part. ^89^Zr long half-life is particularly adapted with mAb kinetic. Nevertheless, this long half-life is not consistent with dosimetry parameters. Even if new TEP allow to inject less activity, patients generally receive higher radiation from ^89^Zr-labeled mAb PET—approximately 0.5 mSv/MBq either 20–40 mSv for 37–74 MBq ^89^Zr [127,128]. So, even if ^89^Zr is particularly interesting for preclinical, Ab biodistribution studies and long-term studies, ^64^Cu, which has a more intermediate period, is more interesting in terms of dosimetry, with a difference of more than a factor of 10 depending on study and antibody [79,129]. Another advantageous property of ^64^Cu is for use as both imaging and therapeutic agents. Due to the unique emission properties of copper isotopes, the use of the theranostic pair copper 64/67 is of great interest due to the convenient interchangeability of copper ions. Within the scope of theranostic approach, the use of β^+^/β^−^ pair radiolabeling, respectively for diagnostic then therapy, with the same mAb is very promising because the same distribution/pharmacokinetic is expected. ImmunoPET allows the determination of the patient dosimetry to optimize the cumulated activity and to predict the therapy response by a quantitative measurement of the tumor-antibody uptake. Another important physical property is positron range. Even if ^64^Cu and ^98^Zr have both interesting properties, the positron range of ^64^Cu is shorter than that of ^89^Zr due to its lower positron energy (Table 1). So, ^64^Cu-PET has a superior spatial resolution to ^89^Zr-PET. Whereas spatial resolution was similar between ^18^F-FDG and ^64^Cu-HCl [130], the spatial resolution and image quality of ^89^Zr PET were inferior to those of ^18^F PET [131].

Among the parameters to consider, costs and availability of isotopes are also very important. Both radionuclides can be produced by a medical cyclotron, but ^64^Cu can also be produced by nuclear reactor. As a result of the growing interest in these 2 radionuclides, production capacities for both radionuclides have increased significantly in recent years. 

Recent advances in clinical trials provide solid evidence to support the potential role of radioactive ^89^Zr and ^64^Cu as useful radiopharmaceuticals for immunoPET and cancer imaging. As interest in ^89^Zr is older, ^89^Zr was superior in the number of clinical studies. Nevertheless, taking into account their period and the resulting dosimetry, ^89^Zr is suitable for a proof-of-concept study, as its half-life (78.4 h) enables the Ab to be studied over a longer kinetic period. In more advanced studies involving injection into humans, the use of ^64^Cu could be more appropriate in order to optimize patient radiation protection thanks to its more intermediate half-life.

## Figures and Tables

**Table 1:** Comparison of Zirconium 89 and Copper 64. EC: electronic capture; E_max_: maximum energy.

	^89^Zr	^64^Cu
**Period**	78.4 h Optimal for intact mAb	12.7 h Optimal for mAb fragment
**Broadcast**	β^+^ (23%) EC (77%) E_max_ β^+^ 897 keV Eγ 909 keV	β^+^ (19%)/β^−^ (40%) EC (41%) E_max_ β^+^ 656 keV Eγ 1346 keV
**Biodistribution**	Affinity of Zr^4+^ for bone (epiphysis) in preclinical studies Transchelation in vivo	Affinity of copper for the liver Transchelation in vivo
**Radiochemistry**	High marking efficiency with macromolecules GMP production Risk of metallic contamination Associated high-energy gamma radiation	Low energy β^+^ High marking efficiency with macromolecules Less than 1% associated gamma radiation Emission β^−^ associated with therapy (in preclinical studies)
